# PARTNER ENGAGEMENT STRATEGIES FOR AN EMBEDDED PRAGMATIC CLINICAL TRIAL: A CASE STUDY

**DOI:** 10.1093/geroni/igad104.2780

**Published:** 2023-12-21

**Authors:** Ann Reddy, Ellen McCreedy, Susan Mitchell, Rosa Baier

**Affiliations:** Brown University School of Public Health, Providence, Rhode Island, United States; Brown University School of Public Health, Providence, Rhode Island, United States; Hebrew SeniorLife, Boston, Massachusetts, United States; Brown University School of Public Health, Providence, Rhode Island, United States

## Abstract

Embedded pragmatic clinical trials (ePCTs) rely on providers, rather than research staff, to implement study protocols. Partnerships need to be carefully managed to ensure a sustainable relationship. This case study describes strategies used by our research team while partnering with a large physician practice on an ePCT to improve advance care planning for people with dementia living in 160 assisted living centers. We focus on engagement between the research and provider teams co-designing and leading the implementation. Communication and project management tools held the teams accountable to decisions and set expectations for time and resources. At project initiation, we discussed the commitment of resources through different phases of the research (planning, executing, and monitoring) and determined the frequency and expected attendance for meetings during each phase. We focused on ensuring the intervention and protocols were feasible and integrated into existing clinical workflows. Regular meetings and continuous communication enabled us to make iterative modifications driven by provider needs and feedback. We used a project management register to track deliverables and document key decisions and next steps. A single point of contact on the research team fielded all inquiries and ensured timely responses to provider partners. ePCTs require a unique and active partnership between researchers and providers. In this case study, communication and project management tools enabled researchers and provider partners to identify and make changes to the ePCT’s intervention design and implementation protocol in real time. This case study provides best practices for management of stakeholder relationships in the ePCT context.

